# Extracorporeal life support prior to left ventricular assist device implantation leads to improvement of the patients INTERMACS levels and outcome

**DOI:** 10.1371/journal.pone.0174262

**Published:** 2017-03-30

**Authors:** David Schibilsky, Christoph Haller, Bruno Lange, Barbara Schibilsky, Helene Haeberle, Peter Seizer, Meinrad Gawaz, Peter Rosenberger, Tobias Walker, Christian Schlensak

**Affiliations:** 1 Department of Thoracic and Cardiovascular Surgery, University Medical Center Tuebingen, Tuebingen, Germany; 2 Department of Anesthesiology and Intensive Care Medicine, University Medical Center Tuebingen, Tuebingen, Germany; 3 Department of Cardiology, University Medical Center Tuebingen, Tuebingen, Germany; Medizinische Hochschule Hannover, GERMANY

## Abstract

**Background:**

The objective of this study was to evaluate the outcome of left ventricular assist device (LVAD) implantation after initial extracorporeal life support (ECLS) in patients with cardiogenic shock and the incidence of post implantation right ventricular failure.

**Methods & results:**

All patients on ECLS therapy for cardiogenic shock prior to LVAD implantation (n = 15) between October 2011 and January 2014 were analyzed. Baseline patient characteristics, as well as detailed pre-operative treatment and postoperative outcome data were collected retrospectively. At time of admission to our unit all patients were classified INTERMACS II or higher (12 [80%] INTERMACS I). Improvement to INTERMACS III temporary cardiac support (TCS) at time of LVAD implantation was successful in 14 patients (93.3%). End-organ function recovered during ECLS support. No patient needed ongoing ECLS or additional right ventricular support after LVAD implantation. Both in-hospital and 30-day mortality was 6.7% (n = 1). The median duration of LVAD support was 687.9 ± 374.5 days. At the end of the study (follow-up 810.7 +/- 338.9 days), 13 (86.7%) patients were alive. The majority of patients (10 [66.7%]) remained on LVAD support. Transplantation could be performed in 1 (6.7%) patient, 2 (13.3%) patients could be successfully weaned.

**Conclusion:**

LVAD implantation in ECLS patients leads to improvement of INTERMACS level to INTERMACS III TCS status. Excellent mid-term survival comparable to true INTERMACS III-IV patients could be shown. ECLS prior to LVAD as a bridge-to-bridge therapy may help to lower mortality in primarily unstable patients.

## Background

The growing expertise of specialized ventricular assist device (VAD) centers led to significant improvement in survival rates in current years. However, mortality remains high, especially in INTERMACS I and II patients[1]. The number of patients who are considered too sick for VAD support and their outcome is unknown. Therefore LVAD implantation should be performed prior to the development of cardiogenic shock[1]. Nevertheless, the best treatment for patients presenting in INTERMACS I or II is still a matter of debate.

In recent times, strategies shift towards isolated left ventricular assist device (LVAD) therapy instead of biventricular support, even in patients in cardiogenic shock[2]. Although isolated left-ventricular support has proven good long-term results[3], early perioperative right-heart failure occurs in 6–40% of these patients [4–6]. Temporary right-heart support using a centrifugal pump (RVAD) can be used as bridge to recovery of the right ventricle or as bridge to transplantation[7]. However, the need of RVAD placement in LVAD patients is associated with high mortality rates, especially if the RVAD placement is delayed to the initial LVAD implantation[8]. Furthermore, right ventricular failure necessitating RVAD support adversely effects successful bridging to transplantation[9].

The objective of this study was to evaluate outcome and survival as well as incidence of right ventricular failure of patients in INTERMACS I/II initially supported with extracorporeal life support (ECLS, i.e. v-a-ECMO) and subsequently transitioned to LVAD.

## Methods

We analyzed all LVAD patients undergoing ECLS treatment and subsequent continuous flow LVAD implantation at our center between October 2011 and January 2014 (n = 15).

In this retrospective cohort study, we analyzed the preoperative characteristics, postoperative course, as well as outcome and survival. In 8 (53.3%) patients, LVAD implantation was performed as bridge to transplant (BTT), 4 (26.7%) patients received the assist device as bridge to decision (BTD) and in 3 (20%) patients a bridge to recovery (BTR) from acute myocarditis or ischemic cardiomyopathy pursued.

ECLS was performed by percutaneous femoral cannulation in all patients. ECLS implantations were performed at bedside on the intensive care unit or in the catheterization laboratory. Transesophageal echocardiography or fluoroscopy was used to ensure that the tip of the venous cannula was positioned in the superior vena cava. A 6 or 7 Fr. distal leg perfusion cannula was routinely implanted during percutaneous ECLS implantation. During support there was no major thromboembolic event or bleeding except from minor bleeding at the cannulation side, which did not require surgical revision. Serial echocardiographic examinations were performed to ensure good biventricular unloading during ECLS.

In a subset of patients underlying ventricular function was assessed with ECLS clamp trials (at least 5 min of pump stop with intermittent flushing) or flow reduction (approximately 1 liter per minute for at least 5 min). Transthoracic echocardiography and pulmonary artery catheter measurements were used additionally. As a structured protocol for the assessment of cardiac function on ECLS was developed during the study period, adequate echocardiographic and hemodynamic data was available in only 9 (60.0%) and 7 (46.7%) patients, respectively.

Laboratory parameters to evaluate end-organ function prior to ECLS and LVAD implantation were compared. Creatinine values of patients on hemodialysis (3 [20.0%]) were excluded.

In all 15 patients, the HeartMate II (Thoratec, Ltd.) left ventricular assist device was implanted using complete sternotomy. Implantation was performed on cardiopulmonary bypass or with a modified ECLS system. The modification of the ECLS system included installation of a hard-shell reservoir, which enabled suction and venting during the operation. The very latest implantations have been performed with those modifications and additional unloading with percutaneous cannulation of the right jugular vein. The assist devices were implanted while the heart was unloaded and beating.

All patients were weaned from extracorporeal circulation (ECC) before starting LVAD support and LVAD support was started at low rounds per minute (rpm). In most cases at the end of the operation we did not use more than the clinically used minimum speed of 8600 rpm. Pump speed was gradually increased during the following days on intensive care under echocardiographic guidance. Final adjustment was performed after extubation and catecholamine weaning, aiming at good left ventricular unloading and, if possible, aortic valve opening.

### Statistical analysis

Prism 6 (GraphPad Software Inc.) was used to perform the statistical analysis and to generate graphs. Values are presented as mean +/- SEM. Scatter Plots present mean, standard deviation and individual values. As the laboratory values were not normally distributed according to D’Agostini & Pearson omnibus normality test, Mann-Whitney test was performed to compare ranks. Differences were considered significant if p < 0.05.

The research project and the retrospective analysis of patient data was reviewed and approved by our local ethic committee (Ethics committee of the University Medical Center Tuebingen, Germany). All data were anonymized before analysis.

## Results

At time of admission to our unit or at time of ECLS implantation at the referring hospital 13 out of 15 (86.7%) patients met INTERMACS level I and 3 met INTERMACS level II criteria (Table 1). The use of ECLS stabilized 14 (93.3%) of these patients achieving INTERMACS III TCS (temporary cardiac support) prior to LVAD implantation.

**Table 1:** Patient characteristics and etiology of heart failure.

Patient #	Age (years)	Etiology of heart failure	INTERMACS (pre ECLS)	ECLS (out of center)
1	61	DCM + Myocarditis	I	-
2	49	Myocardial infarction	I	-
3	41	Cardiac Malignancy	I	-
4	20	Myocarditis	II	-
5	49	DCM	I	-
6	49	Myocardial infarction	II	-
7	56	ICMP	I	-
8	53	Myocarditis	II	-
9	47	DCM	I	-
10	50	ICMP	I	+
11	13	Congenital CMP	I	+
12	56	DCM	I	-
13	45	Myocardial infarction	I	+
14	49	Myocardial infarction	I	+
15	54	Myocardial infarction	I	+

DCM—dilated cardiomyopathy; ICMP—ischemic cardiomyopathy; CMP—cardiomyopathy

The reason for cardiogenic shock was myocardial infarction or decompensated ischemic cardiomyopathy in 5 (33.3%) patients, decompensated dilative cardiomyopathy in 4 (26.7%) patients and acute myocarditis in 2 (13.3%) patients (Table 1).

Out-of-center ECLS implantation was performed in 5 patients (33.3%). Cardiopulmonary resuscitation was necessary in 5 patients (33.3%). The duration of ECLS support prior to LVAD implantation was 7.5 +/- 2.5 days (Table 2).

**Table 2:** Treatment and outcome.

Patient #	Age (years)	INTERMACS (pre ECLS)	ECLS (days)	INTERMACS (pre LVAD)	LVAD (days)	Strategy	Outcome
1	61	I	4	III TCS	1326	BTT	ongoing
2	49	I	5	III TCS	1313	BTT	ongoing
3	41	I	7	II TCS	10	BTD	expired
4	20	II	7	III TCS	311	BTR	weaned
5	49	I	11	III TCS	203	BTD	expired
6	49	II	9	III TCS	1047	BTT	ongoing
7	56	I	7	III TCS	970	BTT	ongoing
8	53	II	10	III TCS	369	BTR	weaned
9	47	I	9	III TCS	685	BTT	ongoing
10	50	I	6	III TCS	683	BTR	ongoing
11	13	I	4	III TCS	239	BTT	transplanted
12	56	I	5	III TCS	655	BTD	ongoing
13	45	I	8	III TCS	632	BTD	ongoing
14	49	I	13	III TCS	531	BTT	ongoing
15	54	I	8	III TCS	501	BTT	ongoing

The outcome was defined at the end of the follow-up period.

BTT—bridge to transplant; BTD—bridge to decision; BTR—bridge to recovery; TCS—temporary cardiac support

**Fig 1:**
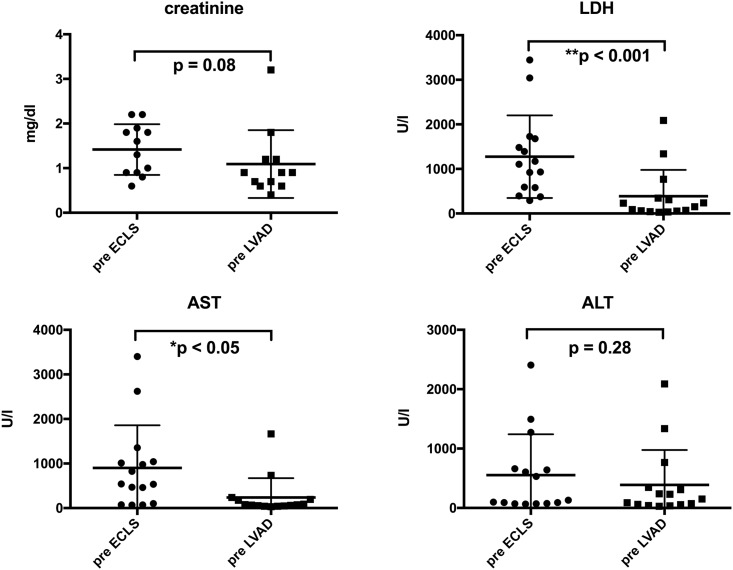
Development of end organ function during ECLS support. The LDH and AST improved significantly from 1275 +/- 239.9 to 389.8 +/- 151.7 U/l (p < 0.001), and 902.9 +/- 246.9 to 239.5 +/- 111.5 U/l (p < 0.005), respectively. The creatinine in patients without hemodialysis (n = 12) decreased insignificantly from 1.42 +/- 0.13 to 1.1 +/- 0.18 mg/dl (p = 0.084). ALT decreased insignificantly from 554.1 +/- 177.2 to 389.8 +/- 151.7 U/l (p = 0.28).

Laboratory values of end organ function recovered during ECLS support (Fig 1). The creatinine of patients without hemodialysis (12 [80.0%]) insignificantly decreased from 1.42 +/- 0.13 to 1.1 +/- 0.18 mg/dl (p = 0.084). The ALT insignificantly decreased from 554.1 +/- 177.2 to 389.8 +/- 151.7 U/l (p = 0.28). The LDH and AST improved significantly from 1275 +/- 239.9 to 389.8 +/- 151.7 (p < 0.001), and from 902.9 +/- 246.9 to 239.5 +/- 111.5 U/l (p < 0.005), respectively.

Prior to LVAD implantation, ECLS weaning trials revealed a left ventricular ejection fraction of 17.3 +/- 6.2%. The right heart function was moderately depressed as indicated by TAPSE of 15 +/- 5.2 mm. The right ventricular geometry was preserved with RV short/long axis ratio of 0.46 +/- 0.1. The invasive hemodynamic measurements revealed a cardiac index of 2.01 +/- 0.32 l/min/m^2^, central venous pressure of 11.9 +/- 4.7 mmHg and wedge pressure of 17.8 +/- 7.7mmHg. Weaning from ECLS or CPB after LVAD implantation was feasible in all patients and there was no right ventricular failure or need of continuing ECLS support.

**Fig 2:**
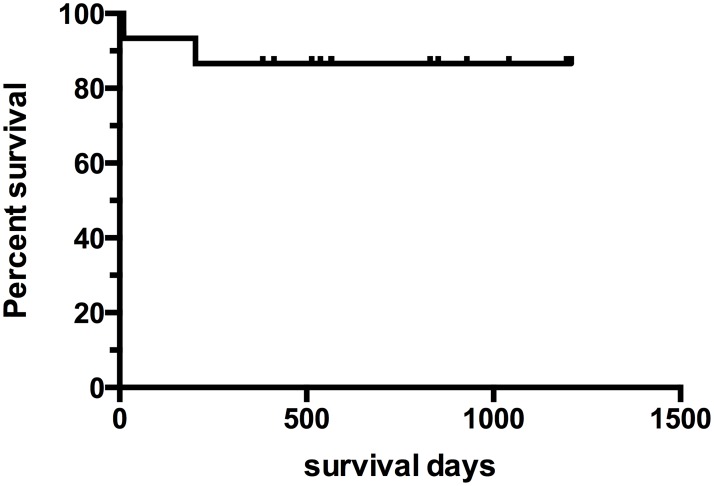
Survival of LVAD patients. The 30-day survival was 93.3%. Hospital discharge was achieved in 93.3%. The median duration of LVAD support was 687.9 (+/- 374.5) days ranging from 1326 to 10 days. At the end of the follow up period 13 (86.7%) of the ECLS bridged patients are still alive. Out of them 10 are on ongoing LVAD support (66.7%), one is transplanted (6.7%) and 2 are successful weaned from LVAD support (13.3%).

The 30-day survival was 93.3% (n = 14). All but one patient (93.3%) could be discharged from the hospital. One patient could not be stabilized, neither by ECLS nor by LVAD and died due to malignant arrhythmia caused by an unknown cardiac malignancy (INTERMACS II TCS implant) on day 10 of LVAD support. At 180 days 14 out of the 15 patients (93.3%) were on ongoing LVAD support (Fig 2).

The median duration of LVAD support was 687.9 (+/- 374.5) days ranging from 10 to 1326 days (Table 2). At the end of the study (follow-up 810.7 +/- 338.9 days) 13 of the ECLS bridged patients are still on ongoing LVAD support, transplanted (1 [6.7%]) or weaned successfully from LVAD (2 [13.3%]).

Supplementary data is shown in the S1 Table (S1 Table).

## Discussion

Our study demonstrates a favorable outcome of LVAD implantation in patients bridged on ECLS. Furthermore, no right heart failure occurred after LVAD implantation.

To our knowledge, this is the largest published cohort of patients bridged from short to long-term mechanical circulatory support (MCS) with detailed mid-term follow up. However, the small number of patients still limits our findings. Unfortunately, we could not assess risk scores for right heart failure prior to ECLS, due to the emergency situations[10,11]. Therefore, we are not able to describe the patient’s individual risk for subsequent right heart failure or compare it to the time of ECLS weaning trials. The values obtained during ECLS weaning trials revealed poor left ventricular but sufficient and sustainable right ventricular recovery. This might be related to sufficient unloading during ECLS support. Unfortunately, we cannot compare our data with suitable control group, as nearly all our INTERMACS I & II patients since 2011 were treated with initial ECLS support prior to LVAD implantation.

The transition from short-term MCS devices to permanent LVADs has previously been described by Mohite et al. with a comparable survival of 6 out of 8 patients at one year[12]. They used relatively long periods of MCS prior to LVAD implantation. The Centrimag device was used as VAD with central cannulation in all cases. Therefore, the circulation was closer to the subsequent VAD circulation and less comparable to veno-arterial ECLS support. Furthermore, the chest was already opened for central cannulation. Obviously, this approach is not suitable for patients, who are critically unstable. In these scenarios, a percutaneous approach at the bedside on the ICU or in the catheterization laboratory is favorable.

One published series describes 9 patients, who were transferred from ECLS to LVAD using a slow transition by decreasing ECMO flow slowly during the first hours after LVAD implantation and while gradually increasing LVAD flow[13]. ECMO had to be used for more than one day after LVAD implantation in 3 patients due to right heart failure. There was one early death and unfortunately no long-term follow up has been reported.

Abdeen et al. analyzed the largest group of patients transferred from ECLS to LVAD support [14]. They analyzed 40 consecutive patients with regard to the use of cardiopulmonary bypass (CPB) during the implantation. Although they did not find a survival advantage of the off-pump group, the survival in both groups was less than 60% at 12 months. The INTERMACS class prior to LVAD implantation is not mentioned. However, one could speculate that LVAD implantation was also performed in patients who could not be stabilized during the ECLS period resulting in unfavorable outcomes.

The recently published cohort from Riebandt et al. reports an outcome comparable to our series[15]. The discharge rate and 1-year survival are 90.9% and 86%. However, within their slightly sicker patient cohort ECLS had to be used after LVAD implantation for 4 +/- 6 days in 68% of patients.

The subsequent use of ECLS and LVAD was also described by Scherer et. al[16]. ECLS was used to treat right ventricular failure after LVAD implantation. The use of ECLS prior to LVAD Implantation was described in 6 of 10 patients. In all cases ECLS needed to be continued after LVAD implantation. The detailed long-term follow-up of these patients is not described but it is mentioned that 40% of all patients died on LVAD support.

In comparison, our approach to wean from ECLS first and subsequently start LVAD flow is a feasible option to protect right ventricular function perioperatively. The direct weaning from ECLS during LVAD implantation has the advantage of easier hemostasis and fewer low flow LVAD events due to competitive ECLS flow.

The unloading of both ventricles during ECLS support ensured by adequate venous cannula position and confirmed by serial echocardiographic examinations during ECLS therapy might be crucial. The days of ventricular unloading during ECLS may give the right ventricle the time to recover from the stress of cardiogenic shock. The preemptive value of this mechanism might be reflected by the avoidance of right ventricular failure in this critically ill patient population. This procedure might be a feasible way to bridge patients to isolated left ventricular support, who would otherwise need biventricular support after cardiogenic shock.

This observational study is limited by its retrospective nature, small patient number and lack of a control group. This is explicable by the fact that almost all INTERMACS I and II patients during the observational period received short-term MCS treatment instead of primary LVAD implantation. The structured assessment of RV function during ECLS weaning trials was developed during the observational period and was therefore only available within a small subset of patients.

## Conclusion

The use of ECLS prior to LVAD implantation results in excellent survival and avoidance of biventricular support in cardiogenic shock patients (INTERMACS I and II). Survival rates are comparable to those seen in patients in INTERMACS III to IV. Therefore, we conclude that patients benefit from initial stabilization to INTERMACS III TCS on ECLS prior to LVAD implantation.

ECLS prior to LVAD implantation as a bridge-to-bridge therapy may help to reduce right heart failure and lower mortality in primarily unstable cardiogenic shock patients.

## Supporting information

S1 Table Study data. De-identified complete dataset.
(XLSX)
